# Development of anti-GPC-1 (glypican-1)-CAR-T cells for adoptive T cell immunotherapies for squamous cell carcinoma

**DOI:** 10.1186/2051-1426-3-S2-P125

**Published:** 2015-11-04

**Authors:** Tomonori Yaguchi, Kenji Morii, Satoshi Serada, Tetsuji Naka, Yutaka Kawakami

**Affiliations:** 1 Division of Cellular Signaling, Institute for Advanced Medical Research, Keio University School of Medicine., Tokyo, Japan; 2 Laboratory of Immune Signal, National Institute of Biomedical Innovation, Osaka, Japan

## 

Immunotherapies using T cells transduced with a chimeric antigen receptor (CAR) gene have been demonstrated as a promising strategy for cancer treatment. CAR-T cells can specifically recognize tumor antigen expressed on the cell surface and eliminate tumors. Although CAR-T cell therapies for hematological malignancies targeting CD19 have recently showed promising clinical outcomes, few success cases have been reported for solid tumors because of the lack of specific cell surface antigens. Glypican-1 (GPC-1) is the member of the glypican family of heparin sulfate proteoglycans that are attached to the cell surface by a glycosylphosphatidylinositol (GPI) anchor. We have previously found GPC-1 were preferentially expressed in squamous cell carcinoma including esophageal cancers, lung cancers, and cervical cancers, and played an important role in cancer growth. We have developed monoclonal antibodies against GPC-1 having *in vivo* anti-tumor effects. In this study, we have developed anti-GPC-1-CAR-T cells and explored their potential for squamous cell carcinoma treatment. The retroviral expression vectors containing the CAR gene consisting of variable regions of the anti-GPC-1 mAb, and CD3ζ/CD28 intracellular signaling domains were generated, and we transduced it into human peripheral blood T cells. The generated anti-GPC-1 CAR-T cells were activated and propagated by IL2 and anti-CD3 Ab. Purified CD8+ anti-GPC-1 CAR-T cells specifically recognized GPC-1 expressed on cancer cells, produced high amount of IFN-γ and TNF-α, and showed high cytotoxic activities. Purified CD4+ anti-GPC-1 CAR-T cells also showed the production of Th1 and Th2 cytokines and cytotoxic activities in a GPC-1-specific manner. These results indicate that GPC1-targeted CAR-T cells have a strong ability to eliminate cancer cells and may be an attractive strategy for treatment of patients with squamous cell carcinoma.

